# Intertwined like a double helix: A meta‐synthesis of the qualitative literature examining the experiences of living with someone with multiple sclerosis

**DOI:** 10.1111/hex.13432

**Published:** 2022-02-04

**Authors:** Anne Parkinson, Crystal Brunoro, Jack Leayr, Vanessa Fanning, Katrina Chisholm, Janet Drew, Jane Desborough, Christine Phillips

**Affiliations:** ^1^ Department of Health Services Research and Policy Australian National University Acton Australia; ^2^ Medical School Australian National University Acton Australia

**Keywords:** care, chronic illness, lived experience, multiple sclerosis, personhood, qualitative

## Abstract

**Background:**

Multiple sclerosis (MS) is a chronic serious condition of uncertain course and outcome. There is relatively little literature on the experiences of people who live with a person with MS. They inhabit a locus of care that spans caring for (a relational act) and caring about (a moral stance, addressing fairness, compassion and justice) the person with MS.

**Methods:**

Using the theoretical lens of personhood, we undertook a scoping review and meta‐synthesis of the qualitative literature on the experiences of people who live with a person with MS, focusing on the nature of, and constraints upon, caring.

**Results:**

Of 330 articles, 49 were included in the review. We identified five themes. One of these—*seeking information and support*—reflects the political economy of care. Two are concerned with the moral domain of care: *caring as labour* and *living with uncertainty*. The final two themes—c*hanging identities* and *adapting to life with a person with MS*—point to the negotiation and reconstitution of personhood for both the person with MS and the people they live with.

**Conclusion:**

People with MS are embedded in relational social networks of partners, family and friends, which are fundamental in the support of their personhood; the people who live with them are ‘co‐constituents of the patient's identity’ assisting them to make sense of their world and self in times of disruption due to illness. Support services and health care professionals caring for people with MS are currently very much patient‐centred; young people in particular report that their roles are elided in the health system's interaction with a parent with MS. There is a need to look beyond the person with MS and recognize the relational network of people who surround them and broaden their focus to encompass this network.

**Patient and Public Involvement:**

Our research team includes four members with MS and two members with lived experience of living or working with people with MS. A third person (not a team member) who lives with a partner with MS provided feedback on the paper.

## 1 BACKGROUND

Multiple sclerosis (MS) is a chronic demyelinating condition of the central nervous system, interfering with nerve impulses within the brain, spinal cord and optic nerves.^1^ More than 2.2 million people are estimated to be affected worldwide.^2^ Although MS can develop at any age, most people are diagnosed between the ages of 20 and 40 years, a life period where many have young families or are about to start a family.^2^

The clinical course of MS varies. The most common form is relapsing–remitting MS, in which periods of stability are interspersed with relapses.^3^ Primary progressive and secondary progressive forms of MS involve gradual worsening of symptoms over time. There is currently no known cure for MS, and the fundamental cause remains unknown.^4^ There are a number of treatment options, but which treatment best suits an individual is uncertain and requires complex decision‐making for patients and families.^4^

Until recently, MS research has tended to focus on clinical outcomes, rather than experiential outcomes, and therefore has tended to elide the lives and experiences of people with MS.^5^, ^6^, ^7^ A chronic disease like MS impacts the whole family, but they have been the subject of little research.^8^, ^9^, ^10^ This points to a gap in the literature on the relational world of people with MS, and how this intersects with care.

In this paper, we are interested in people who live with people with MS. They inhabit a locus of care that spans Noddings' concepts of caring *for* (a relational act) and caring *about* (a moral stance, addressing fairness, compassion and justice).^11^ Not all who live with a person with MS may formally understand their role as caring, but all share their lives with someone with a serious and long‐term illness of uncertain course and outcome. Illnesses like these can lead to people feeling dislocated in biographical time, balancing time for self‐directed projects and time taken up with the body and disease; the people they live with may play important roles in recognizing, supporting and being with the person, devoting time to the management of the body and disease.^12^

For people who live with a person with MS, the relational and moral perspectives of care can be viewed as addressing the recognition and sustaining of personhood.^13^ The purpose of this review was to (i) identify and chart current research knowledge on the experiences of people living with a person with MS and (ii) synthesize these results to explore the enactment of care, and its relation to personhood, by people living with a person with MS.

## 2 METHODS

### 2.1 Theoretical framework

Following Mauss,^14^ we define personhood as a person's human membership, roles or status in society attained through social relations. The lens of personhood is supplemented by a focus on the political economy of care, exploring how social, geographic and professional differentials are evinced in access to and distribution of biomedical knowledge, therapeutic interventions and social supports.^15^, ^16^

### 2.2 Scoping review and meta‐synthesis

We followed the scoping review methodology outlined by Arksey and O'Malley^17^ and enhanced by Levac et al.^18^ It provides a six‐step framework for identifying the research question and relevant studies, selecting studies, charting the data, collating, summarizing and reporting results and consulting relevant stakeholders. Thematic analysis was then used to conduct a meta‐synthesis of the included studies to provide a description of the experiences of people living with a person with multiple sclerosis (PwMS).^19^

### 2.3 Research question

The research question guiding this review was as follows: How do people experience living with a person with MS? Two subquestions were as follows: (i) What are the key experiences of people who live with a person with MS? and (ii) What are the common themes that underpin these experiences?

### 2.4 Identification of studies

Systematic searches were conducted in ProQuest, PubMed, CINAHL and PsychINFO databases of research papers published between 1 January 2003 and 1 January 2021 in English. The final search string used was (‘multiple sclerosis’) AND (experienc* OR perception* OR perspective* OR attitude* OR belief* OR value* OR view*) AND (qualitative OR "focus group*" OR interview* OR narrative*) AND (carer* OR caregiver* OR ‘support person*’ OR parent* OR child* OR brother* OR sister* OR sibling* OR friend* OR family OR families OR partner OR spouse OR husband OR wife). Hand searches were undertaken of the references included in each paper.

### 2.5 Selection of studies

Studies were included if they reported empirical qualitative data about individuals' subjective experiences of living with a person living with MS, were written in English and published in peer‐reviewed journals. Mixed‐method studies were included if qualitative results could be interpreted separately, and studies that included the experience of others (e.g., clinicians, paid carers, people living with MS) were included if the results related to individuals living with people living with MS could be interpreted separately. Reference lists of the included studies were checked. The grey literature was excluded.

### 2.6 Study quality assessment

All included studies were appraised using the Critical Appraisal Skills Programme (CASP) qualitative checklist^20^ by two researchers working independently (A. P., C. B.). Title and abstract screening, and full‐text screening were performed by two reviewers (A. P., C. B.), and conflicts were resolved by a third reviewer (Jane Desborough).

### 2.7 Charting the data

Interpretation and coding began at title and abstract screening and were refined as the data were reviewed. Final coding was performed using NVivo 12 qualitative data analysis software.^21^ Blinded audits of articles were undertaken during analysis to ensure that similar codes and concepts were being applied by two reviewers (A. P., C. B.), and differences were discussed with a third reviewer (Jane Desborough) until consensus was reached. This was followed by a meta‐synthesis and generation of analytical themes that generated new interpretations of the data,^19^, ^22^ framed by key elements of personhood (identity and the social world) and sociopolitical elements of caring.

### 2.8 Patient and public contribution

Patient and public involvement underpins all our research and we have developed longstanding relationships with people in the MS community with whom we work closely, involving them in all aspects of our projects from inception and throughout analysis and preparation of publications. Our research team includes four members with MS and two members with lived experience of living or working with people with MS. They contributed to determining suitable search terms and ongoing discussion of emerging themes throughout the research. A third person (not a team member) who lives with a partner with MS provided feedback on the paper.

## 3 RESULTS

**Figure 1:**
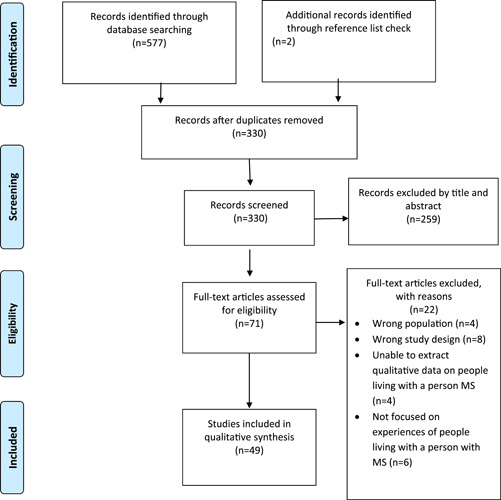
PRISMA flow diagram

The initial search yielded 330 articles, 49 of which were included in the review (Figure 1). The included articles originated from a range of countries (Table 1), and the quality of all studies was considered acceptable on the basis of the CASP tool (Table 2).

**Table 1:** Summary of the included studies

Author (year)	Country	Population	*n*	♀	♂	Age	Aims	Findings	T1	T2	T3	T4	T5
Theme 1 (T1) Seeking information and support													
Theme 2 (T2) Caring as labour													
Theme 3 (T3) Changing identities													
Theme 4 (T4) Living with uncertainty													
Theme 5 (T5) Adapting to life with a person with MS													
Bjorgvinsdottir et al. (2014)	Iceland	Young carers of PwMS	11	6	5	5–18	To study the personal experience of being a young caregiver of a PwMS.	Young carers felt unsupported and isolated. Health professionals need to provide information, support and guidance for young carers.	X	X	X	X	X
Boeije et al. (2003)	The Netherlands	Spouses of PwMS	13	5	8	48–75	To examine how spouses experience caregiving when motivated by a sense of duty.	Health professionals need to recognize that women may be at greater risk of physical and psychological illness and need support.		X	X	X	X
Bogosian et al. (2011)	England	Adolescents of a parent with MS	15	10	5	13–18	To explore how adolescents with a parent with MS adjust to their parents' illness.	Support interventions may be helpful for vulnerable adolescents that consider family and individual factors.	X	X	X	X	X
Bogosian et al. (2009)	England	Partners of PwMS in early stages	15	10	5	32–59	To explore and describe the experiences of partners of PwMS in early stages of MS.	There is a need for health professionals to provide support that focusses on the needs of partners.	X	X	X		X
Boland et al. (2012)	NZ	Partner of PwMS	7	3	4	43–74	To explore how PwMS and their partners cope as a couple.	Clinicians need to be sensitive to the individual coping strategies of both members of a couple.	X	X			X
		PwMS	7	4	3								
Borreani et al. (2014)	Italy	Partner of PwMS	30	16	14	24–91	To identify unmet needs of PwMS living at home with caregivers.	Unmet needs transcended medical issues and embraced psychosocial themes. Lack of support was a critical issue.	X	X	X	X	X
		PwMs	22	14	8	41–77							
		Clinicians	18	11	7	26–59							
Boss and Finlayson (2006)	US	Carer of PwMS	4	2	2	32–69	To explore reactions of family members of a PwMS using power mobility.	Family members had difficulties finding credible sources for information and advice concerning power mobility.		X	X	X	
		PwMS uses mobility aid	11	5	2	31–66							
Bostrom and Nilsagard (2016)	Sweden	Child of PwMS	9	5	4	12–24	To explore issues that are important to acknowledge for a child to cope with a parent with MS.	MS affects the whole family. Health professionals need to support the whole family and empower parents to help children cope.	X	X	X	X	X
		Partner of PwMS	2	1	1								
		PwMS	9	7	2								
Carling et al. (2018)	Sweden	Next of kin of PwMS	20	5	15	26–76	To describe life experiences of next of kin living with a PwMS who falls.	Next of kin are affected by the falls of their cohabiting person. They need practical and emotional support from the health care system.	X	X	X	X	X
Carroll et al. (2016)	UK	Adolescents with MS	15	8	7	9–18	To explore experiences of MS fatigue in the family and how this is managed.	Fatigue is uncontrollable, uncertain and unpredictable and a lack of information exists for PwMS and their carers to manage it.	X	X		X	
		Parents	13	11	2	32–52							
Cheung and Hocking (2004a)	Australia	Spousal carers of PwMS	10	4	6	40–60	To explore lived experiences of spousal carers of PwMS.	Carers' values, concerns and connections with their partner and others shape their way of coping. Health professionals need to provide support and care tailored to their needs.	X	X	X	X	X
Cheung and Hocking (2004b)	Australia	Spousal carers of PwMS	10	4	6	40–60	To explicate the meaning of caring from carers of PwMS.	Caring is a complex emotional relationship of responsibility for carers. They worried about their partners, the future, their own health, institutional care and lack of government support.	X	X		X	
Courts et al. (2005)	US	Spousal carers of PwMS	12	4	8	31–67	To investigate the experiences of spouses of PwMS.	Spouses need information about MS and psycho‐social support. Nurses are well placed to provide information, encourage joining of support groups and offer guidance.	X	X		X	X
Davies et al. (2015)	Wales	Carers of PwSPMS	14	6	8	31–80	To explore experiences of PwMS and their partners in the transition to SPMS.	The process of diagnosing the transition to SPMS was often not transparent and people wanted credible information from health professionals about the transition.	X	X	X	X	
		PwSPMS	20	15	5	31–80							
Dibley et al. (2017)	England	Carers of PwMS	6	4	2	28–76	To understand the impact of bowel dysfunction on PwMS and carers.	Bowel dysfunction impacts the lives of people with MS and their carers. Bowel issues to be discussed more openly, with clinicians instigating a discussion early after MS diagnosis and repeating enquiries regularly.	X	X			
		PwMS	41	31	10	28–76							
du Plooy and Pretorius (2014)	South Africa	Carers of PwMS	8	5	3	28–70	To explore the lived experiences of caregivers of PwMS.	Carer support should be tailored to suit the unique situation of each caregiver and the varied presentation of MS.	X	X	X	X	X
Ebrahimi et al. (2017)	Iran	Carers of PwMS	18	14	4	27–54	To explore the coping strategies of family caregivers of PwMS.	Spiritual connection was key to coping for Iranian family caregivers. Health care providers need to recognize this as they communicate, inform and offer support.	X	X			X
Edmonds et al. (2007)	England	Carers of PwMS	32				To explore the perceptions of PwMS and their carers about their illness and care.	There is a need to better meet patients and carers needs for information, co‐ordination of care and to reduce the feeling of struggling to receive services.	X	X			
		PwMS	6										
Esmail et al. (2010)	Canada	Men with MS	4	4	0	18–60	To understand the experiences of couples' sexual relationship when a male partner has MS.	Clinicians should facilitate open communication, recognize each partner's intimacy needs and assist both partners.	X	X			
		Female partners	4	0	4								
Esmail et al. (2007)	Canada	Women with MS	6	6	0	32–58	To understand the experiences of couples' sexual relationship when a female partner has MS.	Health professionals need to recognize that intimacy needs to be addressed with couples.	X	X	X		X
		Male partners	6	0	6								
Fakolade et al. (2018)	Canada	Carers of PwMS	12	6	6	38–79	To explore shared experiences of physical activity by caregiver/care‐recipient dyads.	Clinicians need to target the health and well‐ being of both the caregiver and the PwMS as an interdependent unit.		X	X	X	X
		PwMS	22	16	6	37–71							
Gafari et al. (2017)	Iran	Carers of PwMS	23				To explore the perceptions of caregivers about caring for a PwMS.	Clinicians need to recognize carers and provide support and information.	X	X		X	X
Hebert et al. (2019)	US	Parents of child with MS	42	40	2		To explore experiences of parents receiving the diagnosis of MS for their child.	Diagnosis of paediatric MS is a difficult time for families where additional support and information is needed.	X				X
Hinton and Kirk (2017)	UK	Parents of child with MS	31	20	11		To explore the experiences of parents living with childhood MS.	Health care professionals need to be sensitive to the role that hope plays in supporting parental coping with childhood MS.	X				
Hinton and Kirk (2015)	UK	Children with MS	21	15	6	8–17	To explore diagnosis experiences of parents of children with MS.	Diagnosis of paediatric MS is a lengthy and uncertain process. Clinicians can aid early diagnosis by listening carefully to parents.	X	X		X	X
		Parents	31	20	11								
Hughes et al. (2013)	UK	Carers of PwMS	48	29	19	17–75	To examine the role of the carer among family members and friends of PwMS.	Self‐identification with the role and label of carer is nuanced, shifting and variable. Health and social care professionals need to understand caregiving identity to provide suitable support.	X	X	X		X
Jonzon and Goodwin (2012)	Canada	Daughters of mothers with MS	4	4	0	19–26	To understand the play experiences of daughters of mothers with MS.	Children of a PwMS experienced guilt and worry as a result of caregiving that matured them beyond their years.		X	X	X	X
Liedstrom et al. (2010)	Sweden	Next of kin to PwMS	44	24	20	19–70	To examine the psychological well‐being of next of kin of PwMS.	Nurses are well placed to start family support groups; nursing interventions that focus on resources for the family of a PwMS are needed.	X	X	X		X
Masoudi et al. (2017)	Iran	Carers of PwMS	14	7	7	20–45	To investigate the experiences of family caregivers of PwMS about stigmatization.	Health care professionals need to provide support for family caregivers of a PwMS to deal with stigma, including teaching social engagement strategies.		X			
Masoudi et al. (2014)	Iran	Carers of PwMS	14	7	7	20–45	To explore the challenges of family caregivers of PwMS.	Emotional and spiritual support for family caregivers is needed.	X		X	X	X
Masterson‐Algar and Williams (2020)	North Wales	Young adults with a parent with MS	14	9	5	16–25	To examine the impact that having a parent with a neurological condition can have on young adults' experiences of growing up.	Young adults reported feelings of abandonment and lack of support from school, peers and services. More initiatives need to be put in place to identify and support these young people.	X	X	X	X	X
Mauseth and Hjalmhult (2016)	Norway	Children of a PwMS	15	8	7	12–18	To examine adolescents' experiences of having a parent with MS.	Adolescents of a PwMS need knowledge about MS, good family functioning and support from health professionals; long‐term intervention programmes offering information and guidance are needed.	X	X		X	X
Mazanderani et al. (2019)	UK	Family/carers of PwMS	60	34	26		To explore the health information work of family members of a PwMS.	When developing information services, it is important that partners and other family members of a PwMS are taken into consideration; clinicians need to find out who the key ‘information worker' is within a family.	X	X			
		PwMS	17	13	4								
McKeown et al. (2004)	Ireland	Carers of PwMS	17	11	6	18–65	To gain an understanding of the experiences of caregivers of PwMS.	Health professionals need to be aware that a caregiver's attitude and acceptance of support with caregiving may change over time; tailored interventions to individual needs are required.	X	X	X	X	X
	(Northern and Republic)												
Moberg et al. (2017)	Denmark	Young adults with a parent with MS	14	12	2	18–25	To explore young adults' experiences of growing up with a parent with MS.	Health care professionals can support the family coping with chronic parental illness by promoting openness and knowledge about the illness. Some of the children need psychological help on a long‐term basis.	X	X	X	X	X
Mutch (2010)	England	Spouses of PwMS	8	4	4	50–74	To understand the experiences of the partner living with and caring for a spouse with MS.	Partners felt obligated to care and a sense of loss as they prioritized the partner. Health professionals need to provide informational and emotional support.		X	X	X	X
Neate et al. (2020)	Australia	Partners of PwMS	21	6	15	20–79	To explore the views of partners of PwMS about the future and how engagement with lifestyle modification may have impacted these views.	Lifestyle modification enabled some partners to develop a sense of empowerment and control, and a subsequent confidence and positivity about their future.		X	X	X	X
	New Zealand												
	UK												
	Switzerland												
Neate et al. (2019b)	Australia New Zealand	Partners of PwMS	21	6	15	20–79	To explore the experiences of partners of PwMS who have adopted lifestyle modification and the impact on the couple's intimate relationship.	Positive relationship benefits were experienced around improved communication and having a greater sense of closeness and feeling more connected.	X	X			X
	UK												
	Switzerland												
Neate et al. (2019a)	Australia New Zealand	Partners of PwMS	21	6	15	28–79	To explore changes made by partners of PwMS to improve well‐being.	A broad range of support from family, friends and health care professionals can assist in making and maintaining lifestyle changes.				X	X
	UK												
	Switzerland												
Neate et al. (2018)	Australia New Zealand	Partners of PwMS	21	6	15	28–79	To explore the experiences of partners of PwMS who have adopted lifestyle modification.	Positive psychological changes were experienced by some partners of PwMS embracing lifestyle modifications including acceptance of MS and adaptation to it.					X
	UK												
	Switzerland												
Nilsagard and Bostrom (2015)	Sweden	Children of PwMS	9	5	4		To explore how health care services can support children of a parent with MS.	Parents with MS should be supported and advised to discuss the situation frequently with their children; forums for discussion should be offered.	X			X	
		Partner of PwMS	5	1	4								
		PwMS	9	7	2								
Rollero (2016)	Italy	Male carers of PwMS	24	0	24	37–68	To explore the experience of male carers of a partner with MS.	Social expectation and gender norms impact male caregivers. Health professionals in particular need to provide tailored support for male spousal caregivers.	X	X	X	X	X
Shapiro et al. (2013)	US	Carers mistreated PwMs	7	4	3	34–85	To explore views on, and disclosure of, mistreatment of PwMS.	There is a need for educational and support groups for caregivers who disclose mistreatment of PwMS.		X	X	X	X
		PwMS	8	5	3								
Sillence et al. (2016)	UK	Carers of PwMS	20	13	7	39–73	To explore what kinds of online support carers of a PwMS prefer.	Carers of PwMS found online support engaging and useful; accounts were most compelling where there was a strong sense of shared identity.	X				
Strickland et al. (2015)	Scotland	Support persons of PwMS	9	5	4	25–80	To explore the experience of the MS diagnosis for the support person.	The uncertainty following diagnosis about the nature and progression of MS left the support person transitioning to that of ‘anticipatory carer’.	X	X	X	X	X
Tehranineshat et al. (2020)	Iran	Family caregivers of PwMS	18	13	5	27–60	To identify family caregivers' experiences at the first hospitalization of their patients.	The psychological and social problems of family caregivers can adversely affect their coping mechanism.	X			X	X
Topcu et al. (2020)	England	Carers of PwMS	12	7	5	30–73	To explore the experiences of carers of PwMS and their quality of life through the use of images and narratives.	The nature of carers' quality of life is complex and whilst mostly negative, some positive aspects ameliorate these.	X	X	X	X	
	UK												
	Cyprus												
Turpin et al. (2008)	Australia	Children of PwMS	8	4	4	7–14	To explore the experiences of children who have a parent with MS.	Parental MS affects the roles and responsibilities of the whole family. Children worry about the well‐being of their parent; social and practical support for the parent could reduce children's anxiety.	X	X	X	X	X
Wawrziczny et al. (2019)	France	Partner of PwMS	6	1	5	29–50	To explore the experiences of couples where one has MS.	Health professionals need to provide early interventions and support for individuals to learn acceptance and for couples to maintain emotional communication.	X	X		X	X
		PwMS	6	5	1								

Abbreviations: PwMS, person with multiple sclerosis; PwSPMS, person with secondary progressive multiple sclerosis.

**Table 2:** CASP (Critical Appraisal Skills Programme) assessment

**Author**	**Year**	**Q1**	**Q2**	**Q3**	**Q4**	**Q5**	**Q6**	**Q7**	**Q8**	**Q9**	**Q10a**	**Q10b**	**Q10c**
Question 1: Are the results valid?													
Question 2: Is a qualitative methodology appropriate?													
Question 3: Was the research design appropriate to address the aims of the research?													
Question 4: Was the recruitment strategy appropriate to the aims of the research?													
Question 5: Was the data collected in a way that addressed the research issue?													
Question 6: Has the relationship between researcher and participants been adequately considered?													
Question 7: Have ethical issues been taken into consideration?													
Question 8: Was the data analysis sufficiently rigorous?													
Question 9: Is there a clear statement of findings?													
Question 10: How valuable is the research?													
a. Discussion of the contribution the study makes to existing knowledge													
b. Identification of new areas where research is necessary													
c. Discussion of how the findings can be transferred to other populations													
Bjorgvinsdottir et al.	2014	Y	Y	y	y	y	y	y	y	Y	Y	N	Y
Boeije et al.	2003	Y	Y	Y	Y	Y	Y	CT	Y	Y	Y	Y	Y
Bogosian et al.	2011	Y	Y	Y	Y	Y	N	Y	Y	Y	Y	Y	Y
Bogosian et al.	2009	Y	Y	Y	Y	Y	Y	Y	Y	Y	Y	Y	Y
Boland et al.	2012	Y	Y	Y	Y	Y	Y	Y	Y	Y	Y	Y	Y
Borreani et al.	2014	Y	Y	Y	Y	Y	N	Y	Y	Y	Y	Y	Y
Boss and Finlayson	2006	Y	Y	Y	Y	Y	N	Y	Y	Y	Y	Y	N
Bostrom and Nilsagard	2016	Y	Y	Y	Y	Y	N	Y	Y	Y	Y	N	Y
Carling et al.	2018	Y	Y	Y	Y	Y	N	Y	Y	Y	Y	Y	Y
Carroll et al.	2016	Y	Y	Y	Y	Y	N	Y	Y	Y	Y	Y	N
Cheung and Hocking	2004a	Y	Y	Y	Y	Y	N	Y	Y	Y	Y	Y	Y
Cheung and Hocking	2004b	Y	Y	Y	Y	Y	N	Y	Y	Y	Y	Y	Y
Courts et al.	2005	Y	Y	Y	Y	Y	N	Y	Y	Y	Y	Y	Y
Davies et al.	2015	Y	Y	Y	Y	Y	Y	Y	Y	Y	Y	Y	Y
Dibley et al.	2017	Y	Y	Y	Y	Y	N	Y	Y	Y	Y	Y	Y
du Plooy and Pretorius	2014	Y	Y	Y	Y	Y	N	Y	Y	Y	Y	Y	Y
Ebrahimi et al.	2017	Y	Y	Y	Y	Y	N	Y	Y	Y	Y	Y	Y
Edmonds et al.	2007	Y	Y	Y	Y	Y	N	Y	Y	Y	Y	N	Y
Esmail et al.	2007	Y	Y	Y	CT	Y	CT	CT	CT	Y	Y	Y	Y
Esmail et al.	2010	Y	Y	Y	CT	Y	CT	CT	CT	Y	Y	Y	Y
Fakolade et al.	2018	Y	Y	Y	Y	Y	Y	Y	Y	Y	Y	Y	Y
Gafari et al.	2017	Y	Y	Y	Y	Y	N	Y	Y	Y	Y	Y	Y
Herbert et al.	2019	Y	Y	Y	Y	Y	N	Y	CT	Y	Y	Y	N
Hinton and Kirk	2015	Y	Y	Y	Y	Y	Y	Y	Y	Y	Y	Y	Y
Hinton and Kirk	2017	Y	Y	Y	Y	Y	Y	Y	Y	Y	y	N	N
Hughes et al.	2013	Y	Y	Y	Y	Y	N	Y	Y	Y	Y	N	y
Jonzon and Goodwin	2012	Y	Y	Y	Y	Y	Y	Y	Y	Y	N	N	Y
Liedstrom et al.	2010	Y	Y	Y	Y	Y	Y	Y	Y	Y	Y	Y	Y
Masoudi et al.	2014	Y	Y	Y	Y	Y	N	Y	Y	Y	Y	Y	Y
Masoudi et al.	2017	Y	Y	Y	Y	Y	N	Y	Y	Y	y	N	Y
Masterson‐Algar and Williams	2020	Y	Y	Y	Y	Y	N	Y	Y	Y	Y	Y	Y
Mauseth and Hjalmhult	2016	Y	Y	Y	Y	Y	N	Y	Y	Y	Y	Y	Y
Mazanderani et al.	2019	Y	Y	Y	Y	Y	N	Y	Y	Y	Y	N	N
McKeown et al.	2004	Y	Y	Y	Y	Y	Y	Y	Y	Y	Y	Y	Y
Moberg et al.	2017	Y	Y	Y	Y	Y	Y	Y	Y	Y	Y	N	Y
Mutch	2010	Y	Y	Y	Y	Y	N	Y	Y	Y	N	N	Y
Neate et al.	2018	Y	Y	Y	Y	Y	Y	Y	Y	Y	Y	Y	Y
Neate et al.	2019a	Y	Y	Y	Y	Y	Y	Y	Y	Y	Y	Y	Y
Neate et al.	2019b	Y	Y	Y	Y	Y	Y	Y	Y	Y	Y	Y	Y
Neate et al.	2020	Y	Y	Y	Y	Y	Y	Y	Y	Y	Y	Y	Y
Nilsagard and Bostrom	2015	Y	Y	Y	Y	Y	Y	Y	Y	Y	Y	Y	CT
Rollero	2016	Y	Y	Y	Y	Y	Y	Y	Y	Y	Y	Y	Y
Shapiro et al.	2013	Y	Y	Y	CT	Y	CT	CT	Y	Y	Y	Y	N
Sillence et al.	2016	Y	Y	Y	Y	Y	CT	Y	CT	Y	Y	Y	N
Strickland et al.	2015	Y	Y	Y	Y	Y	Y	Y	Y	Y	Y	Y	Y
Tehranineshat et al.	2020	Y	Y	Y	Y	Y	Y	Y	Y	Y	Y	N	Y
Turpin et al.	2008	Y	Y	Y	Y	Y	Y	Y	Y	Y	Y	Y	Y
Topcu et al.	2020	Y	Y	Y	Y	Y	N	Y	Y	Y	Y	Y	Y
Wawrziczny et al.	2019	Y	Y	Y	CT	Y	CT	Y	Y	Y	Y	Y	Y

Abbreviations: CT, cannot tell; N, no; Y, yes.

People living with a person with MS included women and men who identified as a partner or spouse of a person with MS, next of kin, support person, child of a parent with MS and parent of a child with MS. Experiences were expressed in relation to two distinct time periods—present day‐to‐day existence and the future. Uncertainty was evident in the experiences of most participants and permeated all aspects of life. The experiences described ranged from positive experiences of empowerment and personal growth to negative experiences of disempowerment—encompassing anger, frustration, guilt and helplessness. The ability of people living with a person with MS to accept, and adapt to, the diagnosis of MS of someone close to them, and adapt their lives and the lives of their family to live with their changed circumstances varied. Their position was not static and ebbed and flowed in line with the symptoms of the person with MS, often mirroring their journey.

Five themes were identified that described the experience of living with a person with MS (Table 3): One of these—*seeking information and support*—reflects the political economy of care. Two are concerned with the moral domain of care: *the labour of care* and *living with uncertainty*. The final two themes—c*hanging identities* and *adapting to life with a person with MS*—point to the negotiation and reconstitution of personhood for both the person with MS and the person they live with.

**Table 3:** Thematic framework

Theme	Related experiences
Seeking information and support	Initial diagnosis of person with MS was a shared and emotionally challenging experience,^23^ further impacted by their own lack of knowledge and negative perspective about MS^24^, ^25^
*(Adults living with a person with MS)*	Dissatisfaction with health care providers,^23^, ^24^, ^25^, ^26^, ^27^, ^28^, ^29^, ^30^, ^31^, ^32^ not knowing where to turn for information,^25^, ^27^, ^33^ role of caregiver not valued^26^, ^27^, ^34^, ^35^
	Need for health care providers to provide information and support for carers,^25^, ^27^, ^29^, ^30^, ^34^, ^36^, ^37^, ^38^, ^39^, ^40^, ^41^, ^42^, ^43^, ^44^ support persons,^23^ partners and^26^, ^31^, ^35^, ^43^, ^44^, ^45^, ^46^, ^47^, ^48^, ^49^, ^50^, ^51^, ^52^ family members^24^, ^25^, ^33^, ^40^, ^50^, ^53^, ^54^
	Participants needed to take on the task of sourcing information elsewhere and accessed the internet,^25^, ^33^, ^34^, ^40^, ^47^, ^50^, ^55^, ^56^, ^57^, ^58^ books,^29^, ^33^, ^47^, ^50^, ^55^, ^58^ peer group support networks,^29^, ^40^, ^50^, ^55^, ^57^, ^58^ forums,^50^, ^55^, ^57^ blogs,^50^, ^55^, ^57^ media,^33^, ^40^, ^50^, ^55^ friends,^34^, ^47^, ^50^, ^55^ family,^50^, ^55^ relevant literature^52^ and MS associations^29^, ^47^, ^50^, ^56^, ^57^; information gathering was a valued role often undertaken by the partner/family member.^50^
Seeking information and support	Dissatisfaction with health care providers,^24^, ^27^, ^28^, ^30^, ^32^ not knowing where to turn for information,^27^, ^33^ role of caregiver not valued^26^, ^27^
*(Children of a parent with MS)*	Need for health care providers to provide both information and support for children of parents with MS^24^, ^27^, ^28^, ^30^, ^33^, ^54^, ^59^, ^60^
	Received little information from health care providers^24^, ^27^, ^28^, ^30^; believed health care providers did not understand how the diagnosis affected them^60^
	No opportunity to discuss the illness with health care providers or within a support group led to confusion and fear^27^
	Valued having a clinician talk to them (children) about MS, receiving age‐appropriate written materials and being referred to family group information sessions^33^; accompanying a parent to a treatment session was helpful^30^, ^33^
	Desiring information from parents—believed parents uninformed,^24^, ^27^, ^30^ parents did not understand how the diagnosis that affected their child and^60^ children better able to adjust when parents were open and informative about MS^33^, ^60^
	Lack of support at school,^28^ which made it difficult to manage school workloads, increased potential dropping out, teachers did not understand about living with a chronically ill parent and^27^, ^59^ some adolescents received support from their school nurse^30^
Seeking information and support	Initial diagnosis was distressing,^55^ further impacted by their own lack of knowledge and negative perspective about MS and implications for future potential disability of their child^61^
*(Parents of a child with MS)*	Parents could not rely on health care providers and sourced information elsewhere from the internet,^55^, ^56^ books,^55^ peer group support networks,^55^ forums,^55^ blogs,^55^ media,^55^ friends and family and^55^ MS associations^56^
	Difficulty getting diagnosis as paediatric‐onset MS not well recognized, not being heard; opinion of parents not valued, lack of information and support tailored to needs of children,^32^, ^55^, ^56^, ^61^ hearing the diagnosis at the same time as their child/no warning was disempowering^56^
	Positive experiences included being consulted about sharing diagnosis with the child, being supported/listened to, being provided with age‐appropriate resources and connecting parents to support groups^61^
Caring as labour	Caring incorporates emotional support, personal care, physical care, household tasks and advocacy^62^
*(Adults living with a person with MS)*	Reasons for taking on the role included obligation,^40^, ^43^, ^44^, ^48^, ^62^, ^63^ duty,^62^, ^64^ as part of commitment to marriage,^34^, ^43^ sacrifice,^30^, ^35^, ^36^, ^37^, ^44^, ^48^, ^51^, ^62^, ^65^, ^66^, ^67^ intertwined nature of the relationship between the person with MS and the person living with them^23^, ^45^, ^47^, ^50^, ^51^, ^68^
	Specific symptoms to manage (balance and falling,^37^ bladder and bowel dysfunction,^38^, ^65^ mood swings,^47^, ^66^, ^69^ sexual dysfunction,^48^, ^49^ fatigue^26^, ^39^, ^63^)
	Challenging aspects reported by partner,^26^, ^29^, ^34^, ^35^, ^36^, ^38^, ^39^, ^40^, ^43^, ^44^, ^45^, ^46^, ^47^, ^51^, ^58^, ^63^ carer,^27^, ^31^, ^35^, ^37^, ^42^, ^46^, ^51^, ^58^, ^65^, ^69^ parent of a child with MS and^32^, ^55^ family member^24^, ^37^, ^53^, ^68^
	Recognition of positive experiences supporting a partner,^35^, ^43^, ^47^, ^52^, ^66^ family member^53^
	Men believed that they were less suited to the caring role than women^44^, ^47^, ^65^; some women agreed^47^, ^65^
	Challenges identified included time‐consuming and hard work,^26^, ^31^, ^34^, ^35^, ^37^, ^39^, ^43^, ^44^, ^46^, ^51^, ^62^, ^63^, ^65^, ^68^ it is a ‘full time job’,^39^, ^44^, ^62^, ^63^ extra household tasks increasingly taken on,^34^, ^35^ those who worked found it progressively more demanding,^63^ constant need to plan ahead for any activity was taxing,^26^, ^35^, ^37^, ^44^, ^51^ responsibility of decision‐making was a burden,^68^ acting on their behalf was a burden,^50^ having to provide intimate care was difficult and confronting^38^
	Some participants felt unacknowledged and not valued by health care providers,^26^, ^34^, ^35^ the community and^35^, ^62^ the PwMS^65^
	Changes in the nature of the relationship were challenging and occurred in the early stages of MS,^23^, ^26^ sexual relationship,^48^, ^49^ as the condition progressed and physical/mental capacities were affected^35^, ^37^, ^38^, ^43^, ^44^, ^46^, ^47^, ^53^, ^62^, ^63^
Caring as labour	Reasons for taking on the role included obligation^27^, ^59^, ^60^, ^64^ and recognition of the intertwined nature of their relationship with their parent with MS^59^
*(Children of a parent with MS)*	Specific symptoms to manage included bladder and bowel dysfunction,^24^ mood swings and^24^ fatigue^24^, ^30^, ^64^
	Challenging aspects of caring reported^27^, ^30^, ^54^, ^59^, ^60^, ^64^
	Recognition of positive aspects of supporting a parent^59^, ^60^
	Challenges identified included time‐consuming and hard work,^24^, ^27^, ^34^, ^59^ children tired from extra household responsibilities,^27^, ^54^, ^59^, ^64^ constant need to plan ahead for any activity was taxing and^24^ having to provide intimate care was difficult and confronting^27^, ^54^
	Some children felt unacknowledged and not valued by health care providers, or the community, or their parent with MS^27^, ^28^, ^30^, ^64^
Changing identities	Hard to maintain sense of self in the face of changing roles, expectations and identity as the person with MS transitions from healthy partner or family member to one needing support^23^, ^26^, ^35^, ^62^
*(Adults living with a person with MS)*	Not all identify as a ‘carer’ believing the label limiting,^23^, ^62^ especially in the early stages, when transitioning to becoming a future or ‘anticipatory’ carer^23^
	Role of ‘carer’ shifting and variable over time, adopted to differing degrees^62^
	MS changed their partner, the dynamics of their relationship and themselves^43^, ^53^, ^63^, ^65^
	Loss of a partner, a friend, a lover or a coparent, who was replaced by a person who needed ongoing support^35^, ^44^
	Loss of self and status as a person with paid employment and one valued by society,^35^, ^37^, ^58^, ^63^ becoming invisible as family, friends or health care providers ask only about the PwMS^37^
	Loss of connectedness with friends, family and work colleagues as social lives became significantly reduced,^24^, ^26^, ^34^, ^36^, ^37^, ^39^, ^41^, ^42^, ^44^, ^48^, ^53^, ^58^, ^63^, ^68^ lack of spontaneity due to need for constant planning,^26^, ^35^, ^37^, ^43^, ^44^, ^58^ foregoing shared activities with partner,^24^, ^26^, ^34^, ^37^, ^48^, ^58^, ^63^, ^66^, ^67^ people feel uncomfortable when a person is in a wheelchair,^35^ access to homes and public places difficult with a wheelchair,^37^, ^42^, ^44^, ^58^, ^68^ some independence restored when the PwMS obtained a mobility aid^68^
Changing identities	Role of parent/child reversed, with the child acting as a parent^59^, ^64^
	Parenting themselves and younger siblings^27^, ^54^, ^60^
*(Children of a parent with MS)*	Did not identify with others the same age as more mature/different priorities^59^, ^64^
	Becoming invisible as family, friends or health care, providers ask only about the person with MS^24^, ^27^, ^54^, ^60^
	Reduced social life, lack of spontaneity and less time with friends^24^
	Shaped/changed every aspect of their lives and identity^28^
Living with uncertainty	Four key uncertainty time points were identified: during diagnosis, in daily life, during social or clinical interactions and when thinking about the future^55^
*(Adults living with a person with MS)*	Unpredictable trajectory of MS a constant source of worry for partners,^43^, ^44^, ^46^, ^47^, ^51^, ^58^, ^68^, ^70^ carers,^25^, ^34^, ^35^, ^36^, ^39^, ^42^, ^58^, ^65^ support persons,^23^ parents of person with MS and^32^, ^55^ next of kin^37^
	Uncertainty was psychologically distressing for partners who described apprehension, uncertainty and guilt around not knowing how to help the PwMS^66^
	Carers worried about getting sick and being unable to care for the PwMS,^36^, ^42^, ^46^ with many reporting anxiety and depression^31^, ^36^, ^41^, ^63^, ^65^, ^66^
Living with uncertainty	Uncertain future caused constant worry^24^, ^28^, ^30^, ^33^, ^54^, ^59^, ^60^, ^64^
	Children experienced anxiety and depression^27^, ^30^, ^54^, ^60^
	MS diagnosis overwhelming and worry inducing, often continuing into adulthood^27^, ^28^, ^60^
*(Children of a parent with MS)*	Some children concealed feelings, worried about burdening parents,^28^, ^59^, ^60^ some did not talk about it as it was not openly discussed within the family^27^
	Adolescents of a parent with MS reported being constantly worried about how the MS might progress,^24^, ^28^, ^30^ whether their parent was having a ‘bad day’,^30^ whether the parent with MS had hurt themselves/needed assistance^24^, ^54^, ^60^
	Some adolescents recognized they had become overprotective; worried continually whenever they were away,^64^ felt they could not spend time with friends,^54^, ^59^ were delaying leaving home,^64^ feeling guilty if they left to pursue their education^28^, ^30^
Adapting to life with a person with MS	Acceptance,^23^, ^26^, ^37^, ^40^, ^45^, ^48^, ^66^ including children of a parent with MS^24^, ^30^ and normalization as MS was gradually accepted into their family life, creating a new normality.^24^, ^59^
*(Adults living with a person with MS)*	Strong spiritual beliefs,^25^, ^39^, ^40^ hope for a cure or for slow disease progression,^26^, ^40^, ^45^, ^66^ including for a parent,^30^ comfort that MS was not a condition they feared might be worse for partner,^45^ parent^59^ or child.^61^
	Establish positive relationships with like‐minded people who are supportive^52^
	Positive, supportive and nurturing relationships with health care providers for both the person with MS and partner highly valued, abandon negative influences such as health care providers who are unhelpful/unsupportive^52^
	Not focus solely on MS, but concentrate on living as normal a life as possible,^23^, ^24^, ^44^, ^59^ putting practical strategies in place for managing everyday life as needed, acquiring equipment to facilitate routine tasks and adapting living spaces to enable independence^23^, ^24^, ^26^, ^31^, ^37^, ^44^, ^53^, ^63^; some parents of children with MS chose to withdraw from support groups that acted as a constant reminder of MS^55^
	Some partners/carers chose to distance themselves from the person with MS, and^35^, ^46^, ^51^, ^63^ for others the emotional cost of caregiving was overwhelming and they railed at the unfairness of life and their helplessness; for some, this anger led to resentment of the person with MS and intentional mistreatment^65^
	It was important for participants to maintain their health and well‐being and to take time out for themselves, have a break from their stressful environment and socialize with friends^24^, ^36^, ^37^, ^43^, ^48^, ^52^, ^62^, ^65^, ^66^, ^67^; being able to speak to someone honestly about their situation helped to release emotional tension.^23^, ^26^, ^47^, ^51^, ^65^
	Support outside of immediate family reduced after the initial diagnosis of the person with MS, leaving participants feeling isolated^26^, ^35^, ^36^, ^40^, ^41^, ^42^, ^44^
	Support and encouragement provided by families enabled couples to adjust to change^52^
	Positive experiences of personal growth and empowerment were reported by some participants who found that even though they might have been placed in situations outside their comfort zone, they learned how to be adaptable, face challenges and develop new skills,^35^, ^40^, ^44^, ^47^, ^48^, ^60^, ^66^, ^70^, ^71^ connect and communicate better with their partner^70^ and reassess their lives to determine what they most valued^66^
	Some reassessed their lives and determined what they valued most, often appreciating and finding greater meaning in life, becoming less concerned about material things and prioritizing family over work^52^, ^66^
	Working together as a couple to achieve goals including better communication^70^
	Some valued and embraced their role as a carer, feeling a sense of pride and accomplishment, or seeing themselves as an expert, or in one case, taking on the role of campaigning for people with MS^62^
Adapting to life with a person with MS	Some children of people with MS were ashamed or embarrassed about their parents' condition and did not want to discuss it with their friends or let other people know the degree to which they cared for them^24^, ^27^, ^28^, ^30^, ^41^, ^54^, ^60^
*(Children of a parent with MS)*	Adolescents reported that they found respite by changing focus and listening to music, playing computer games, spending time with friends^30^, ^54^
	Social support from friends and family members was also a key enabler for children; it helped them to better adjust to a parent's MS by providing not only practical help in everyday tasks but also emotional support^27^, ^28^, ^30^, ^54^, ^59^, ^64^
	Some adolescents received support from their school nurse or doctor^30^
	Some young caregivers developed personal stability and inner strength by following the rules set down for their friends with normal family lives, which enabled them to create order, discipline and control in their own lives^27^
	Later in life, some young caregivers believed that it was support from their life partners that enabled them to move on and deal with their situation^27^
	Some learned how to be adaptable, face challenges and develop new skills^28^, ^35^, ^40^, ^44^, ^47^, ^48^, ^52^, ^60^, ^66^
	Young adults were also able to experience personal growth by acting as a caregiver, which built confidence, independence and resilience^28^, ^30^, ^59^, ^60^, ^64^; many young adult carers choose to pursue health‐related education stemming from their empathy for people with chronic illness and having to be responsible and organized from an early age^60^

Abbreviation: MS, multiple sclerosis; PwMS, person with multiple sclerosis.

### 3.1 Seeking information and support

Carers reported being excluded from a care ecosystem organized around the health care provider and the person with MS. Partners,^26^ carers of persons transitioning to secondary progressive MS and^34^ parents of children with MS, especially while in pursuit of a diagnosis,^55^, ^56^, ^61^ reported feeling ignored and undervalued by clinicians who did not treat them as equal partners in the health care relationship. Young carers of a parent with MS noted, in particular, that they were marginalized by clinicians whose focus was solely on the parent.^27^, ^28^

The initial diagnosis was described by partners and parents of people with MS as being a shared and emotionally challenging experience.^23^, ^55^ This was compounded by lack of knowledge about MS and inaccurate or negative preconceptions about MS, including expectations of severe disability.^24^, ^25^, ^61^ Most studies identified pressing needs for clinicians to provide informational, practical and emotional support for carers,^25^, ^27^, ^29^, ^30^, ^34^, ^36^, ^37^, ^38^, ^39^, ^40^, ^41^, ^42^, ^43^, ^44^ support persons,^23^ partners^26^, ^31^, ^35^, ^43^, ^44^, ^45^, ^46^, ^47^, ^48^, ^49^, ^50^, ^51^ and family members,^24^, ^25^, ^33^, ^40^, ^50^, ^53^, ^54^ including parents^32^, ^55^, ^56^, ^61^ and children of people with MS.^24^, ^27^, ^28^, ^30^, ^33^, ^54^, ^59^, ^60^

In this setting of knowledge asymmetry, information gathering became a valued activity undertaken by partners or family members of a person with MS.^50^ In their search for knowledge, people living with a person with MS accessed information from multiple sources outside the health system, including the internet,^25^, ^33^, ^34^, ^40^, ^47^, ^50^, ^55^, ^56^, ^57^, ^58^ books,^29^, ^33^, ^47^, ^50^, ^55^, ^58^ peer support networks,^29^, ^40^, ^50^, ^55^, ^57^, ^58^ forums,^50^, ^55^, ^57^ blogs,^50^, ^55^, ^57^ media,^33^, ^40^, ^50^, ^55^ friends,^34^, ^47^, ^50^, ^55^ family,^50^, ^55^ relevant literature^52^ and MS associations.^29^, ^47^, ^50^, ^56^, ^57^

### 3.2 Caring as labour

Most studies described the challenging aspects of a caring role when living with a person with MS—as a partner,^26^, ^29^, ^34^, ^35^, ^36^, ^38^, ^39^, ^40^, ^43^, ^44^, ^45^, ^46^, ^47^, ^51^, ^63^ informal carer,^27^, ^31^, ^35^, ^37^, ^42^, ^46^, ^51^, ^58^, ^65^, ^69^ parent,^32^, ^55^ child^27^, ^30^, ^54^, ^59^, ^60^, ^64^ or family member^24^, ^37^, ^53^, ^68^ of a person with MS. Caring activities included, but were not limited to, providing emotional support, personal/intimate care, physical care, household tasks and advocacy.^62^ Care was represented as a positive activity by some, enabling personal growth by carers supporting a partner,^35^, ^43^, ^47^, ^58^, ^66^ parent^59^, ^60^ or family member^53^ with MS.

Many studies also noted that care was experienced as a form of moral labour, marked by the interdigitation of domestic and health needs of persons with MS and the person living with them.^23^, ^45^, ^47^, ^50^, ^51^, ^59^, ^68^ Participants felt an obligation to care for the person with MS,^40^, ^43^, ^44^, ^48^, ^62^, ^63^ including their young and older children.^27^, ^59^, ^60^, ^64^ Participants in some studies described care as a duty,^62^, ^64^ part of their commitment to marriage^34^, ^43^ or a sacrifice.^30^, ^35^, ^36^, ^37^, ^44^, ^48^, ^51^, ^62^, ^65^, ^66^, ^67^ Some participants, including children of persons with MS,^27^, ^28^, ^30^, ^64^ felt unacknowledged and that their role as a caregiver was not valued by health care practitioners,^26^, ^27^, ^34^, ^35^ the community^35^, ^62^, ^64^ or the person with MS.^30^, ^65^ Several studies reported gender disparities, with men believing that they were less suited to a caring role than women^44^, ^47^, ^65^; some women agreed.^47^, ^65^

### 3.3 Living with uncertainty

Uncertainty about MS diagnosis, treatment, prognosis and management underpinned the experiences and concerns of people living with a person with MS. The unpredictable trajectory of MS was presented as a constant source of worry for partners,^43^, ^44^, ^46^, ^47^, ^51^, ^58^, ^68^, ^70^ carers,^25^, ^34^, ^35^, ^36^, ^39^, ^42^, ^65^ support persons,^23^ children^24^, ^28^, ^30^, ^33^, ^54^, ^58^, ^59^, ^60^, ^64^ and parents of people with MS,^25^, ^32^, ^55^ and their next of kin.^37^ This existential uncertainty was psychologically distressing for partners who described not knowing how to help the person with MS^66^ and for carers worried about being unable to care for them if they themselves were unwell.^27^, ^36^, ^42^, ^46^ Faced with an uncertain future, participants in many studies reported experiencing anxiety and depression,^31^, ^36^, ^41^, ^58^, ^63^, ^65^, ^66^ including children.^27^, ^28^, ^30^, ^54^, ^60^ The MS diagnosis was overwhelming and worrying for children, and often continued into adulthood.^27^, ^60^ Some children worried about burdening their parents, and described concealing their feelings and making efforts to reassure them that they were coping.^59^, ^60^

### 3.4 Changing identities

Four studies described the struggle of people living with a person with MS to maintain their sense of self in the face of changing roles, as the person with MS transitioned from healthy partner or family member to one needing support.^23^, ^26^, ^35^, ^62^ While people carried out a wide range of caring activities, not everyone identified as a ‘carer’, some preferring instead to be recognized according to their relationship (e.g., partner, sister, mother),^62^ especially in the early stages when transitioning to becoming a future or ‘anticipatory’ carer.^23^ The role of ‘carer’ could shift and vary over time.^62^ Some spouses and partners reported a shift in their sense of self as being individual—characterized by independence, strength and freedom—to being intertwined, subsumed by caring and support of the person with MS. Many experienced a sense of loss^35^, ^44^; they felt that MS had changed their partner, the dynamics of their relationship and themselves.^43^, ^53^, ^63^, ^65^ Those who had to stop or reduce their work sometimes experienced a loss of self and status.^35^, ^37^, ^58^, ^63^ Some children acting as caregiver to a parent with MS described a reversal of the parent–child role,^59^, ^64^ with others reporting taking on parental obligation for younger siblings as well as parenting themselves.^27^, ^54^, ^60^ Many experienced a loss of connectedness with friends, family and work colleagues as their social lives contracted.^24^, ^26^, ^34^, ^36^, ^37^, ^39^, ^41^, ^42^, ^44^, ^48^, ^53^, ^58^, ^63^, ^68^ Participants—particularly children of people with MS^24^, ^27^, ^54^, ^60^—described a sense of becoming invisible as family, friends or health care providers never asked about them, only about the person with MS.^24^, ^37^

Few studies focused on gendered perspectives or gender roles of people living with a person with MS. Four studies about the experiences of spousal caregivers considered the differing perspectives of women and men in the context of their sexual relationship and changing roles,^48^, ^49^ and ability to care more broadly.^44^, ^63^ One study found that women give care and take on more responsibilities in response to societal expectations, whereas men believed that they were going beyond such expectations when they give care.^63^ Only one study specifically examined the experiences of adult male partners caring for a person with MS in relation to gender norms and the difficulties that they faced showing vulnerability and seeking support.^44^

### 3.5 Adapting to life with a person with MS

People who live with people with MS face a need to develop a long‐term management approach to the challenges to identity, living with uncertainty and the moral demands of care. People used various adaptation strategies. Acceptance was viewed as the first step,^23^, ^26^, ^37^, ^40^, ^45^, ^48^, ^66^ including for children.^24^, ^30^ Spiritual beliefs helped some,^25^, ^39^, ^40^ whereas others focused on hope for a cure or slow disease progression.^26^, ^40^, ^45^, ^66^ Many made a conscious effort to live in the present rather than dwelling on the future,^34^, ^35^, ^37^, ^39^, ^45^, ^53^, ^57^ and many resolved to not focus solely on MS, but concentrate on living as normal a life as possible.^23^, ^24^, ^26^, ^31^, ^37^, ^44^, ^53^, ^63^ In contrast, some partners or carers chose to distance themselves from the person with MS.^35^, ^46^, ^51^, ^63^ In one study, the emotional cost of caregiving led to resentment and intentional mistreatment.^65^ The most valued coping strategy was maintaining personal well‐being and taking time out for themselves,^24^, ^36^, ^37^, ^43^, ^48^, ^58^, ^62^, ^65^, ^66^, ^67^ and speaking to someone about their situation to relieve emotional tension.^23^, ^26^, ^28^, ^47^, ^51^, ^58^, ^65^ Social support from friends and family members was a key enabler for children of a person with MS.^27^, ^28^, ^30^, ^54^, ^59^, ^64^

Some participants found that even though they might have been placed in situations outside their comfort zone, they learned to be adaptable, face challenges and develop new skills,^35^, ^40^, ^44^, ^47^, ^48^, ^60^, ^66^, ^70^, ^71^ connect and communicate better with their partner^70^ and reassess their lives to determine what they most valued.^66^ Others learned to value and embrace their role as a carer, feeling a sense of pride and accomplishment.^62^ Young adults also experienced personal growth through acting as a caregiver of a parent with MS, which built confidence, independence and resilience,^28^, ^30^, ^59^, ^60^, ^64^ often leading them to health‐related education and professions later in life.^60^

## 4 DISCUSSION

People with chronic illness are embedded in relational social networks of partners, family and friends, which are fundamental in the support of the personhood of people with MS; they are ‘co‐constituents of the patient's identity’, assisting them to make sense of their world and self in times of disruption.^72^

The five themes described in this review shed light on the intertwined experiences of people living with a person with MS as a partner, spouse, child, parent, family member or next of kin and the person with MS. Irrespective of their relationship with the person with MS and the severity of the condition, their experiences have commonalities. The two themes *living with uncertainty* and *caring as labour* were present across most studies. These two themes were integral to *changing identities* and drivers of *seeking information and support* and *adapting to life with a person with MS*.

The theme *seeking information and support* highlights the role taken on by people living with a person with MS in advocating and addressing asymmetries of knowledge. There is a particular urgency with MS, where early diagnosis and treatment is key to better outcomes,^73^ and many people take on the role of advocacy early when the person with MS may be overwhelmed or too physically unwell to undertake this role themselves.^12^ This underpins the critical need for information and practical and emotional support highlighted across the studies, as decisions about treatment options affect both the person with MS and those close to them. These ‘decision partners’ play a vital role in shared decision‐making about treatment.^74^ The literature included in our review indicates that people living with a person with MS, including families, are often treated as minor players by health care practitioners, resonating with previous research about chronic illness management.^75^, ^76^

The impact of chronic disease on family members is profound and spreads across all aspects of life including for both children^77^ and parents. Young carers are both the most vulnerable and the most likely to be ignored within the health system. As demonstrated in the themes *caring as labour* and *changing identities*, caring can greatly influence the life course of young people both physically and psychologically, affecting their education, economic opportunities, friendships and social support networks.^78^ Health care practitioners need to be aware of situations where a young person may provide most of the care to a parent, as this is an emotional burden and can severely affect them.^79^ They may need ongoing support, especially to manage priorities, including school workloads.^79^, ^80^ An Australian study about services for younger carers recommended that support for families should be a policy priority so that young people do not have to take on roles that are disruptive to their own development, functioning and education.^81^

Purcal and colleagues^78^ propose an analytical framework that aims to assist young carers to seek support and relieve tension, while at the same time working to mitigate their caring responsibilities, with the ultimate goal of preventing their entrenchment in a caring role. The enactment of this framework seeks to facilitate *adapting to life with a person with MS* and it removes the assumption that this adaptation necessarily incorporates a caring role. One related resource designed to support young carers is Talk‐Link, an Australian‐based service that offers telephone counselling and access to peers for different age groups over an 8‐week period.^78^, ^82^ Schools are also well placed to provide support for young carers and offer programmes to develop understanding among teachers and peers.^79^ The experiences described by those who live with, care for and support a person with MS closely mirror those described by people with MS.^15^ Our results suggest that the negotiation and support of the personhood is a mutual process between the person living with them and the person themselves. The lives of the caregiver and the care recipient can mirror one another, with both losing autonomy, the caregiver through the acceptance of responsibility and the care recipient through needing care.^83^ In effect, the lives of carer or family member, and the person with MS are intertwined, forming what has been described as a ‘double helix’, with their needs being ‘largely inseparable’.^84^ The contexts in which these experiences occurred were not siloed; rather, like life and families, they were a melange of overlapping experiences spread throughout work, school, social and home life and seeking health care.

The experiences described in the theme *living with uncertainty* indicate that uncertainty is an ongoing challenge for people living with a person with MS and aligns with previous research indicating that uncertainty is a key feature of family members' experiences of chronic conditions, including progressive neurological illness.^85^, ^86^

Tams and colleagues^86^ identify four dimensions where uncertainty is experienced by families of those affected by MS: initial ambiguity about the diagnosis; the typically uncertain diagnosis; the unpredictability of illness course; and how family roles and relationships might be affected over time as MS progresses. These dimensions align with the findings of our review, which also identified two distinct illness‐related temporal dimensions to uncertainty that people living with a person with MS must tackle: the present and the future. There are different challenges for decision‐making and planning in each of these time periods.^85^ For example, physical symptoms such as fatigue, mobility and balance issues, and side effects of treatments for the person with MS have practical implications for daily living that may impact planning any or all activities, especially when symptoms are erratic.^15^ Future concerns, however, are more focused on disease progression, reduced mobility, ability to have a family, diminishing mental capacity, reduced ability to work and potential economic impacts,^15^ which, for people who live with a person with MS, may be framed as concerns about ‘anticipated caring’.^23^

Rather than eliminate uncertainty, Tams and colleagues^86^ suggest that families can benefit from learning to live with, tolerate and adapt to it. They encourage clinicians working with people affected by progressive neurological conditions (including MS) to use strength‐based interventions to help families manage illness‐related uncertainty and live well in the face of uncertainty.

Gregory^87^ argues that tasks and activities related to caring and chronic disease management are family practices that form part of the ‘ongoing lived experiences of the family relationship’. Furthermore, it is through the incorporation of, and adaptation to, new ways of living into family practices that a feeling of continuity and normality is achieved.^87^ This is especially reflected in the themes *living with uncertainty* and *adapting to life with a person with MS*, where our review found that participants described a sense of normalization as MS was adapted into their everyday lives, creating a ‘new normal’ in family life, which enabled them to adapt. All five themes identified in our review may be seen to contribute to disruption of ontological security for the person living with a person with MS, creating many life challenges. Participants in studies included in our review described finding difficulty maintaining social connections, seeking emotional and psychological support, living in the present and making sense of their world.

The absence of studies addressing the gendered experiences of people caring for people with MS is striking. The broader caring literature, particularly that from a feminist perspective,^83^ highlights the fact that caring is predominantly performed by women, and that this has a political dimension, with caring being an undervalued example of ‘invisible work’. In the case of MS, which affects many more women than men,^88^ the carer roles are disproportionately taken by men—or at least, the household membership includes high numbers of women with MS and male partners. Expectations of unpaid labour contributed by partners who are outside of, or have left, the paid workforce may play out differently for male family members than for female family members. The lack of research on male carers was also raised in an earlier review of the experiences of spousal caregivers of a person with MS.^89^ However, male participants in the included studies represent those who stayed to act as caregivers, potentially excluding partners who had chosen to leave. There have been mixed findings in studies on partner abandonment among women with MS, with a US study finding female gender of the person with MS to be a strong predictor of male partners leaving the relationship,^90^ while studies from Denmark^91^ and Sweden^92^ found that it was not. Further research in this area is needed to better understand the caring work done by men and how it relates to ideas of masculinity and paid and unpaid work. Research encompassing the gendered experiences of parents of children with MS and children of parents with MS is also needed to address this knowledge gap.

## 5 LIMITATIONS

A strength of our study is the inclusion of quality appraisal, which is not a requirement of scoping review approaches.^17^ A potential limitation may be the exclusion of quantitative studies, which often provide greater sample sizes and uniformity. This synthesis does not include non‐peer‐reviewed literature such as personal narratives or reports from advocacy groups. Only studies written in English were included, which resulted in few studies from non‐western countries with less developed health care systems, and that may have different cultural expectations of and influences on families.

## 6 CONCLUSION

Both the person with MS and the household or family member caring for or living with them often work together in mutual support of personhood. Adapting to life with a person with MS is challenging and requires learning to live with uncertainty and find ways of making sense of one's world through integrating MS into everyday family life and practices. Support services and health care practitioners are currently very much centred on the individual patient. They need to look beyond the person with MS and recognize the relational network of people who surround them and shift their focus to become family‐ or household‐centred. There is a need to design interventions that involve and support the active engagement of decision partners in health care decision‐making related to chronic disease, benefitting decision partners, clinicians and patients. Future research and policy foci that are currently underexplored include the experiences of young carers or household family members of people with MS and gendered expectations and performance of carer roles among people living with a person with MS.

## CONFLICT OF INTERESTS

The authors declare that there are no conflict of interests.

## AUTHOR CONTRIBUTIONS

Anne Parkinson contributed to conceptualization, methodology, validation, formal analysis, investigation and data curation, writing—original draft and review and editing, visualization and project administration. Crystal Brunoro contributed to conceptualization, methodology, validation, formal analysis, investigation, data curation and writing—review and editing. Jack Leayr contributed to formal analysis and writing—review and editing. Vanessa Fanning contributed to writing—review and editing. Katrina Chisholm  contributed to writing—review and editing. Janet Drew contributed to writing—review and editing. Jane Desborough contributed to conceptualization, methodology, investigation, writing—review and editing, supervision and funding acquisition. Christine Phillips contributed to conceptualization, methodology, writing—review and editing, and supervision.

## Data Availability

All data generated or analysed during this study are included in this published article and its Supporting Information files.

## References

[1] MS Australia What is MS? 2017. Accessed August 1, 2020. https://www.msaustralia.org.au/what-ms

[2] GBD 2016 Multiple Sclerosis Collaborators . Global, regional, and national burden of multiple sclerosis 1990–2016: a systematic analysis for the Global Burden of Disease Study 2016. Lancet Neurol. 2019;18(3):269‐285. 10.1016/S1474-4422(18)30443-5 30679040 PMC6372756

[3] Thompson AJ , Baranzini SE , Geurts J , Hemmer B , Ciccarelli O . Multiple sclerosis. Lancet. 2018;391(10130):1622‐1636. 10.1016/S0140-6736(18)30481-1 29576504

[4] Reich DS , Lucchinetti CF , Calabresi PA . Multiple sclerosis. N Engl J Med. 2018;378(2):169‐180. 29320652 10.1056/NEJMra1401483 PMC6942519

[5] The Editor . Patient‐reported outcomes in the spotlight. Lancet Neurol. 2019;18(11):981. 10.1016/S1474-4422(19)30357-6 31609205

[6] Gehr S , Kaiser T , Kreutz R , Ludwig WD , Paul F . Suggestions for improving the design of clinical trials in multiple sclerosis‐results of a systematic analysis of completed phase III trials. EPMA J. 2019;10(4):425‐436. 10.1007/s13167-019-00192-z 31832116 PMC6883016

[7] Celius EG , Thompson H , Pontaga M , et al. Disease progression in multiple sclerosis: a literature review exploring patient perspectives. Patient Pref Adher. 2021;15:15‐27. 10.2147/PPA.S268829 PMC7802794 33447018

[8] Eriksson E , Wejåker M , Danhard A , Nilsson A , Kristofferzon M‐L . Living with a spouse with chronic illness—the challenge of balancing demands and resources. BMC Public Health. 2019;19:19. 10.1186/s12889-019-6800-7 31014309 PMC6480606

[9] Golics CJ , Basra MKA , Finlay AY , Salek S . The impact of disease on family members: a critical aspect of medical care. J R Soc Med. 2013;106(10):399‐407. 10.1177/0141076812472616 23759884 PMC3791092

[10] Golics CJ , Basra MKA , Salek MS , Finlay AY . The impact of patients' chronic disease on family quality of life: an experience from 26 specialties. Int J Gen Med. 2013;6:787‐798. 10.2147/IJGM.S45156 24092994 PMC3787893

[11] Noddings N . Caring: A Feminine Approach to Ethics and Moral Education. University of California Press; 1984.

[12] Monks J , Frankenberg R . Disability and culture. In: Ingstad B , Reynolds Whyte S , eds. Being Ill and Being Me: Self, Body, and Time in Multiple Sclerosis Narratives. Berkley: University of California Press; 1995:107‐134.

[13] Buch E . Senses of care: Embodying inequality and sustaining personhood in the home care of older adults in Chicago. Am Ethnol. 2013;40(4):637‐650. 10.1111/amet.12044 26401062 PMC4577066

[14] Mauss M . A category of the human mind. In: Carrithers M , Collins S , Lukes S , eds. The Category of the Person: Anthropology, Philosophy, History. Cambridge University Press; 1985:1‐25.

[15] Desborough J , Brunoro C , Parkinson A , et al. ‘It struck at the heart of who I thought I was’: a meta‐synthesis of the qualitative literature examining the experiences of people with multiple sclerosis. Health Expect. 2020;23(5):1007‐10027. 10.1111/hex.13093 32578287 PMC7696124

[16] Browne P , Chandraratna D , Angood C , et al. Atlas of multiple sclerosis 2013: a growing global problem with widespread inequity. Neurology. 2014;83(11):1022‐1024. 10.1212/WNL.0000000000000768 25200713 PMC4162299

[17] Arksey H , O'Malley L . Scoping studies: towards a methodological framework. Int J Soc Res Method. 2005;8(1):19‐32. 10.1080/1364557032000119616

[18] Levac D , Colquhoun H , O'Brien KK . Scoping studies: advancing the methodology. Implement Sci. 2010;5(1):69. 10.1186/1748-5908-5-69 20854677 PMC2954944

[19] Thomas J , Harden A . Methods for the thematic synthesis of qualitative research in systematic reviews. BMC Med Res Methodol. 2008;8(1):45. 10.1186/1471-2288-8-45 18616818 PMC2478656

[20] Critical Appraisal Skills Programme CASP Qualitative checklist. 2018. Accessed August 1, 2020. https://casp-uk.net/casp-tools-checklists/2018

[21] NVivo . Qualitative Data Analysis Software [computer program]. Version 12 QSR International Pty Ltd. 2020. https://www.qsrinternational.com/nvivo/nvivo-products/nvivo-12-plus

[22] Braun V , Clarke V . Using thematic analysis in psychology. Qual Res Psychol. 2006;3(2):77‐101. 10.1191/1478088706qp063oa

[23] Strickland K , Worth A , Kennedy C . The experiences of support persons of people newly diagnosed with multiple sclerosis: an interpretative phenomenological study. J Adv Nurs. 2015;71(12):2811‐2821. 10.1111/jan.12758 26337059

[24] Bostrom K , Nilsagard Y . A family matter—when a parent is diagnosed with multiple sclerosis. A qualitative study. J Clin Nurs. 2016;25(7‐8):1053‐1061. 10.1111/jocn.13156 26868176

[25] Tehranineshat B , Yektatalab S , Momennasab M , Bijani M , Mohammadi F . The experiences of multiple sclerosis patients' family caregivers at the first hospitalization of their patients: a qualitative study. Patient Prefer Adher. 2020;14:1159‐1172. 10.2147/ppa.S257746 PMC7367720 32764889

[26] Bogosian A , Moss‐Morris R , Yardley L , Dennison L . Experiences of partners of people in the early stages of multiple sclerosis. Mult Scler. 2009;15(7):876‐884. 10.1177/1352458508100048 19168601

[27] Bjorgvinsdottir K , Halldorsdottir S . Silent, invisible and unacknowledged: experiences of young caregivers of single parents diagnosed with multiple sclerosis. Scand J Caring Sci. 2014;28(1):38‐48. 10.1111/scs.12030 23550661

[28] Masterson‐Algar P , Williams S . “Thrown Into the Deep End”: mapping the experiences of young people living in a family affected by a neurological condition. Qual Health Res. 2020;30(5):717‐729. 10.1177/1049732319900498 31994448

[29] Edmonds P , Vivat B , Burman R , Silber E , Higginson IJ . ‘Fighting for everything’: service experiences of people severely affected by multiple sclerosis. Mult Scler. 2007;13(5):660‐667. 10.1177/1352458506071789 17548447

[30] Mauseth T , Hjalmhult E . Adolescents' experiences on coping with parental multiple sclerosis: a grounded theory study. J Clin Nurs. 2016;25(5‐6):856‐865. 10.1111/jocn.13131 26762177

[31] Gafari S , Khoshknab MF , Nourozi K , Mohamadi E . Informal caregivers' experiences of caring of multiple sclerosis patients: a qualitative study. Iran J Nurs Midwifery Res. 2017;22(3):243‐247. 10.4103/1735-9066.208168 28706551 PMC5494956

[32] Carroll S , Chalder T , Hemingway C , Heyman I , Moss‐Morris R . “It feels like wearing a giant sandbag.” Adolescent and parent perceptions of fatigue in paediatric multiple sclerosis. Eur J Paediatr Neurol. 2016;20(6):938‐945. 10.1016/j.ejpn.2016.06.004 27422092

[33] Nilsagard Y , Bostrom K . Informing the children when a parent is diagnosed as having multiple sclerosis. Int J MS Care. 2015;17(1):42‐48. 10.7224/1537-2073.2013-047 25741226 PMC4338642

[34] Carling A , Nilsagård Y , Forsberg A . ‘You are just left to get on with it’: qualitative study of patient and carer experiences of the transition to secondary progressive multiple sclerosis. BMJ Open. 2015;5(7):940‐947. 10.1136/bmjopen-2015-007674 PMC4513516 26201723

[35] Cheung J , Hocking P . The experience of spousal carers of people with multiple sclerosis. Qual Health Res. 2004;14(2):153‐166. 10.1177/1049732303258382 14768455

[36] du Plooy DR , Pretorius C . Unmet needs of people with severe multiple sclerosis and their carers: qualitative findings for a home‐based intervention. PLoS One. 2014;9(10):e109679‐369. 10.1371/journal.pone.0109679 25286321 PMC4186842

[37] Carling A , Nilsagard Y , Forsberg A . Making it work: experience of living with a person who falls due to multiple sclerosis. Disabil Rehabil. 2018;2017:1‐8. 10.1080/09638288.2018.1514078 30299167

[38] Dibley L , Coggrave M , McClurg D , Woodward S , Norton C . “It's just horrible”: a qualitative study of patients' and carers' experiences of bowel dysfunction in multiple sclerosis. J Neurol. 2017;264(7):1354‐1361. 10.1007/s00415-017-8527-7 28550483

[39] du Plooy DR , Pretorius C . The caregiver experience: a South African perspective on caring for people with multiple sclerosis. J Psychol Afr. 2014;24(4):361‐369.

[40] Ebrahimi H , Hasankhani H , Namdar H , Khodadadi E , Fooladi M . Dealing with chronic illness: experiences of Iranian families of persons with multiple sclerosis—a qualitative study. Mult Scler Int. 2017;2017:9243161‐9243165. 10.1155/2017/9243161 29082042 PMC5610797

[41] Masoudi R , Khayeri F , Rabiei L , Zarea K . A study of stigma among Iranian family caregivers of patients with multiple sclerosis: a descriptive explorative qualitative study. Appl Nurs Res. 2017;34:1‐6. 10.1016/j.apnr.2016.11.012 28342617

[42] McKeown LP , Porter‐Armstrong AP , Baxter GD . Caregivers of people with multiple sclerosis: experiences of support. Mult Scler. 2004;10(2):219‐230. 10.1191/1352458504ms1008oa 15124770

[43] Mutch K . In sickness and in health: experience of caring for a spouse with MS. Br J Nurs. 2010;19(4):214‐219. 10.12968/bjon.2010.19.4.46782 20220670

[44] Rollero CP . The experience of men caring for a partner with multiple sclerosis. J Nurs Scholarsh. 2016;48(5):482‐489. 10.1111/jnu.12231 27391528

[45] Boland P , Levack WM , Hudson S , Bell EM . Coping with multiple sclerosis as a couple: ‘peaks and troughs’—an interpretative phenomenological exploration. Disabil Rehabil. 2012;34(16):1367‐1375. 10.3109/09638288.2011.645115 22256892

[46] Cheung J , Hocking P . Caring as worrying: the experience of spousal carers. J Adv Nurs. 2004;47(5):475‐482. 10.1111/j.1365-2648.2004.03126.x 15312110

[47] Courts NF , Newton AN , McNeal LJ . Husbands and wives living with multiple sclerosis. J Neurosci Nurs. 2005;37(1):20‐27. 10.1097/01376517-200502000-00004 15794441

[48] Esmail S , Huang J , Lee I , Maruska T . Couple's experiences when men are diagnosed with multiple sclerosis in the context of their sexual relationship. Sex Disabil. 2010;28(1):15‐27. 10.1007/s11195-009-9144-x

[49] Esmail S , Munro B , Gibson N . Couple's experience with multiples sclerosis in the context of their sexual relationship. Sex Disabil. 2007;25(4):163‐177. 10.1007/s11195-007-9054-8

[50] Mazanderani F , Hughes N , Hardy C , Sillence E , Powell J . Health information work and the enactment of care in couples and families affected by multiple sclerosis. Sociol Health Illn. 2019;41(2w):395‐410. 10.1111/1467-9566.12842 30677163

[51] Wawrziczny E , Corrairie A , Antoine P . Relapsing‐remitting multiple sclerosis: an interpretative phenomenological analysis of dyadic dynamics. Disabil Rehabil. 2019;41:1‐9. 10.1080/09638288.2019.1617794 31131646

[52] Neate SL , Taylor KL , Jelinek GA , De Livera AM , Brown CR , Weiland TJ . Taking active steps: changes made by partners of people with multiple sclerosis who undertake lifestyle modification. PLoS One. 2019;14(2):e0212422. 10.1371/journal.pone.0212422 30817765 PMC6394935

[53] Liedstrom E , Isaksson AK , Ahlstrom G . Quality of life in spite of an unpredictable future: the next of kin of patients with multiple sclerosis. J Neurosci Nurs. 2010;42(6):331‐341. 10.1097/jnn.0b013e3181f8a5b2 21207771

[54] Turpin M , Leech C , Hackenberg L . Living with parental multiple sclerosis: children's experiences and clinical implications. Can J Occup Ther. 2008;75(3):149‐156. 10.1177/000841740807500306 18615926

[55] Hinton D , Kirk S . Living with uncertainty and hope: a qualitative study exploring parents' experiences of living with childhood multiple sclerosis. Chronic Illn. 2017;13(2):88‐99. 10.1177/1742395316664959 27539955

[56] Hinton D , Kirk S . Paediatric multiple sclerosis: a qualitative study of families' diagnosis experiences. Arch Dis Child. 2015;100(7):623‐945. 10.1136/archdischild-2014-306523 25552262

[57] Sillence E , Hardy C , Briggs P , Harris PR . How do carers of people with multiple sclerosis engage with websites containing the personal experiences of other carers and patients? J Health Inform. 2016;22(4):1045‐1054. 10.1177/1460458215607938 26460102

[58] Topcu G , Buchanan H , Aubeeluck A , Ülsever H . Informal carers' experiences of caring for someone with multiple sclerosis: a photovoice investigation. Br J Health Psychol. 2020;26:1363‐1374. 10.1111/bjhp.12482 33128428

[59] Bogosian A , Moss‐Morris R , Bishop FL , Hadwin J . How do adolescents adjust to their parent's multiple sclerosis? An interview study. Br J Health Psychol. 2011;16(2):430‐444. 10.1348/135910710X521492 21489068

[60] Moberg JY , Larsen D , Brødsgaard A . Striving for balance between caring and restraint: young adults' experiences with parental multiple sclerosis. J Clin Nurs. 2017;26(9‐10):1363‐1374. 10.1111/jocn.13587 27648554

[61] Hebert D , Geisthardt C , Hoffman H . Insights and recommendations from parents receiving a diagnosis of pediatric multiple sclerosis for their child. J Child Neurol. 2019;34(8):464‐471. 10.1177/0883073819842420 31012369

[62] Hughes N , Locock L , Ziebland S . Personal identity and the role of ‘carer’ among relatives and friends of people with multiple sclerosis. Soc Sci Med. 2013;96:78‐85. 10.1016/j.socscimed.2013.07.023 24034954 PMC3778435

[63] Boeije HR , Van , Doorne‐Huiskes A . Fulfilling a sense of duty: how men and women giving care to spouses with multiple sclerosis interpret this role. Community Work Fam. 2003;6(3):223‐244. 10.1080/1366880032000143438

[64] Jonzon AJ , Goodwin DL . Daughters of mothers with multiple sclerosis: their experiences of play. Adapt Phys Activ Q. 2012;29(3):205‐223. 10.1123/apaq.29.3.205 22811563

[65] Shapiro J , Wiglesworth A , Morrison EH . Views on disclosing mistreatment: a focus group study of differences between people with MS and their caregivers. Mult Scler Relat Disord. 2013;2(2):96‐102. 10.1016/j.msard.2012.09.006 25877630

[66] Neate SL , Taylor KL , Jelinek GA , De Livera AM , Brown CR , Weiland TJ . Pyschological shift in partners of people with multiple sclerosis who undertake lifestyle modification: an interpretive phenomenological study. Front Psychol. 2018;9(15):348‐358. 10.3389/fpsyg.2018.00015 29445346 PMC5797767

[67] Fakolade A , Lamarre J , Latimer‐Cheung A , Parsons T , Morrow SA , Finlayson M . Understanding leisure‐time physical activity: voices of people with MS who have moderate‐to‐severe disability and their family caregivers. Health Expect. 2018;21(1):181‐191. 10.1111/hex.12600 28722772 PMC5750693

[68] Boss TM , Finlayson M . Responses to the acquisition and use of power mobility by individuals who have multiple sclerosis and their families. Am J Occup Ther. 2006;60(3):15. 10.5014/ajot.60.3.348 16776403

[69] Masoudi R , Abedi HA , Abedi P , Mohammadianinejad SE . Iranian family caregivers' challenges and issues in caring of multiple sclerosis patients: a descriptive explorative qualitative study. Iran J Nurs Midwifery Res. 2014;19(4):416‐423. 25183985 PMC4145499

[70] Neate SL , Taylor KL , Jelinek GA , et al. On the path together: experiences of partners of people with multiple sclerosis of the impact of lifestyle modification on their relationship. Health Soc Care Comm. 2019;27(6):1515‐1524. 10.1111/hsc.12822 PMC6851851 31368624

[71] Neate SL , Taylor KL , Nag N , et al. Views of the future of partners of people with multiple sclerosis who attended a lifestyle modification workshop: a qualitative analysis of perspectives and experiences. Int J Environ Res Public Health. 2020;18(1):85. 10.3390/ijerph18010085 PMC7796062 33374429

[72] van Nistelrooij I , Visse M , Spekkink A , de Lange J . How shared is shared decision‐making? A care‐ethical view on the role of partner and family. J Med Ethics. 2017;43(9):637‐644. 10.1136/medethics-2016-103791 28356489

[73] Cerqueira JJ , Compston DAS , Geraldes R , et al. Time matters in multiple sclerosis: can early treatment and long‐term follow‐up ensure everyone benefits from the latest advances in multiple sclerosis? J Neurol Neurosurg Psychiatry. 2018;89(8):844‐850. 10.1136/jnnp-2017-317509 29618493 PMC6204938

[74] Gray TF , Nolan MT , Clayman ML , Wenzel JA . The decision partner in healthcare decision‐making: a concept analysis. Int J Nurs Stud. 2019;92:79‐89. 10.1016/j.ijnurstu.2019.01.006 30743199

[75] Whitehead L , Jacob E , Towell A , Abu‐Qamar M , Cole‐Heath A . The role of the family in supporting the self‐management of chronic conditions: a qualitative systematic review. J Clin Nurs. 2018;27(1‐2):22‐30. 10.1111/jocn.13775 28231630

[76] Bužgová R , Kozáková R . Informing patients with progressive neurological disease of their health status, and their adaptation to the disease. BMC Neurol. 2019;19(1):250. 10.1186/s12883-019-1488-y 31653233 PMC6815047

[77] Jahanshahi M , Sheikh S . Long‐term neurological illness in parents has a substantial impact on the lives of children. Br J Neurosci Nurs. 2007;3(6):246‐252. 10.12968/bjnn.2007.3.6.23708

[78] Purcal C , Hamilton M , Thomson C , Cass B . From assistance to prevention: categorizing young carer support services in Australia, and international implications. Soc Pol Admin 2012;46(7):788‐806. 10.1111/j.1467-9515.2011.00816.x

[79] Chikhradze N , Knecht C , Metzing S . Young carers: growing up with chronic illness in the family—a systematic review 2007‐2017. J Compassionate Health Care. 2017;4(1):12. 10.1186/s40639-017-0041-3

[80] Pakenham KI , Cox S . Effects of benefit finding, social support and caregiving on youth adjustment in a parental illness context. J Child Fam Stud. 2018;27(8):2491‐2506. 10.1007/s10826-018-1088-2

[81] Joseph S , Sempik J , Leu A , Becker S . Young carers research, practice and policy: an overview and critical perspective on possible future directions. Adolesc Res Rev. 2020;5(1):77‐89. 10.1007/s40894-019-00119-9

[82] Carers NSW Australia Talk‐Link. 2021. Accessed February 2, 2021. https://www.carersnsw.org.au/news-events/events/counselling/

[83] Rummery K , Fine M . Care: a critical review of theory, policy and practice. Soc Pol Admin. 2012;46(3):321‐343. 10.1111/j.1467-9515.2012.00845.x

[84] Mintz SG . The double helix: when the system fails the intertwined needs of caregiver and patient. Health Aff. 2014;33(9):1689‐1692. 10.1377/hlthaff.2013.1305 25201673

[85] Webster M . The cycle of uncertainty: parents' experiences of childhood epilepsy. Sociol Health Illn. 2019;41(2):205‐218. 10.1111/1467-9566.12815 30353551 PMC6849525

[86] Tams R , Prangnell SJ , Daisley A . Helping families thrive in the face of uncertainty: strengths based approaches to working with families affected by progressive neurological illness. Neurorehabilitation. 2016;38(3):257‐270. 10.3233/nre-161317 27030902

[87] Gregory S . Living with chronic illness in the family setting. Sociol Health Illn. 2005;27(3):372‐392. 10.1111/j.1467-9566.2005.00447.x 15953213

[88] Sellner J , Kraus J , Awad A , Milo R , Hemmer B , Stüve O . The increasing incidence and prevalence of female multiple sclerosis—A critical analysis of potential environmental factors. Autoimmun Rev. 2011;10(8):495‐502. 10.1016/j.autrev.2011.02.006 21354338

[89] Appleton D , Robertson N , Mitchell L , Lesley R . Our disease: a qualitative meta‐synthesis of the experiences of spousal/partner caregivers of people with multiple sclerosis. Scand J Caring Sci. 2018;32(4):1262‐1278. 10.1111/scs.12601 30144143

[90] Glantz MJ , Chamberlain MC , Liu Q , et al. Gender disparity in the rate of partner abandonment in patients with serious medical illness. Cancer. 2009;115(22):5237‐5242. 10.1002/cncr.24577 19645027

[91] Pfleger CCH , Flachs EM , Koch‐Henriksen N . Social consequences of multiple sclerosis. Part 2. Divorce and separation: a historical prospective cohort study: Clinical and Laboratory Research. Mult Scler. 2010;16(7):878‐882. 10.1177/1352458510370978 20483882

[92] Landfeldt E , Castelo‐Branco A , Svedbom A , Löfroth E , Kavaliunas A , Hillert J . The long‐term impact of multiple sclerosis on the risk of divorce. Mult Scler Relat Disord. 2018;24:145‐150. 30007180 10.1016/j.msard.2018.07.002

